# Quantitative proteomics analysis reveals the tolerance of wheat to salt stress in response to *Enterobacter cloacae* SBP-8

**DOI:** 10.1371/journal.pone.0183513

**Published:** 2017-09-06

**Authors:** Rajnish Prakash Singh, Ashish Runthala, Shahid Khan, Prabhat Nath Jha

**Affiliations:** 1 Department of Biological Science, Birla Institute of Technology and Science (BITS), Pilani, Rajasthan, India; 2 Indian Institute of Science, Bangalore, India; Huazhong University of Science and Technology, CHINA

## Abstract

Salinity stress adversely affects the plant growth and is a major constraint to agriculture. In the present study, we studied the role of plant growth promoting rhizobacterium (PGPR) *Enterobacter cloacae* SBP-8 possessing ACC deaminase activity on proteome profile of wheat (*Triticum aestivum* L.) under high salinity (200 mM NaCl) stress. The aim of study was to investigate the differential expressed protein in selected three (T-1, T-2, T-3) treatments and absolute quantification (MS/MS analysis) was used to detect statistically significant expressed proteins. In this study, we investigated the adaptation mechanisms of wheat seedlings exposed to high concentration of NaCl treatment (200 mM) for 15 days in response to bacterial inoculation based on proteomic data. The identified proteins were distributed in different cellular, biological and molecular functions. Under salt stress, proteins related to ion-transport, metabolic pathway, protein synthesis and defense responsive were increased to a certain extent. A broader comparison of the proteome of wheat plant under different treatments revealed that changes in some of the metabolic pathway may be involved in stress adaption in response to PGPR inoculation. Hierarchical cluster analysis identified the various up-regulated/down-regulated proteins into tested three treatments. Our results suggest that bacterial inoculation enhanced the ability of wheat plant to combat salt stress via regulation of transcription factors, promoting antioxidative activity, induction of defense enzymes, lignin biosynthesis, and acceleration of protein synthesis.

## Introduction

Soil salinity is a major problem in the agriculture sector that inhibits the crop growth and productivity. It is estimated that around 20% of cultivated land, and up to 50% of all irrigated land are severely affected by high salinity effects worldwide. Most of this salt-affected land has arisen from the accumulation of salts over long periods of time in arid and semiarid zones [1]. The irrigation with salinized water and scarce rain fall contribute to further increased salt stress that leads to decrease in crop productivity [2]. Increased salt concentrations in the soil decrease the ability of a plant to take up water from the ground, whereas the increased level of Na^+^ and Cl^-^ negatively affect growth by impairing metabolic processes and decreasing photosynthetic efficiency. Besides this, many of the components of signaling pathways have also been affected in plant responses to salinity inferred by transcriptomics and reverse genetics approaches [3]. Plant's response to salinity is a complex phenomenon which involves activation or modification of processes occurring at physiological, biochemical, and molecular levels [4]. Various injuries induced by salt stress are controlled by a chain of gene expression and several proteomic changes. A detailed identification and analyses of changes occurring at the protein level in a salt stressed-crop seems to be a rational approach for understanding the molecular mechanism of response to salt. Coping with salt stress involves complicated mechanisms that include developmental, morphological, physiological, and biochemical strategies [5]. Further, salt stress-regulation genes are expressed in abundance which leads to the changes in total protein profile that help plants to adapt to salt accumulation [6].

Despite the fact that the generation of the salt-resistant genetically modified plants (GMP) are promising [7–9], they are not very popular among users firstly, due to ethical issues and secondly, due to low public acceptance. Thus, GMPs have not been widely applied at the field level. Uses of the plant growth promoting rhizobacteria (PGPR) with 1-aminocyclopropane-1-carboxylate (ACC) deaminase activity have been reported to improve plant growth at high salt concentrations [10,11]. The development of proteomics tools has greatly facilitated the application of plant protein analysis to investigate plant-rhizobacteria interaction. Many of the plant proteins expressed in response to beneficial interactions provide resistance to pathogens and abiotic stress, and are also helpful for plant growth promotion [12–13]. Previous studies [14–15] have shown that certain plant growth promoting bacteria like *Pseudomonas putida* UW4 and *Pseudomonas fluorescens* counteract the salinity stress effects in *Brassica napus* and *Oryza sativa* by a differential expression of proteins related to plant defense, energy metabolism, photosynthesis, protein degradation and oxidative stress response.

Wheat (*Triticum aestivum* L.), the second most important food crop, has high nutritional value and is rich in protein, minerals, calcium, iron, riboflavin and vitamin-A, etc. [16]. The proteins in wheat seeds can be divided into albumins, globulins, gliadins, and glutenins that are important enzymes in plant growth [17]. Few previous studies have been conducted on wheat to reveal the molecular mechanism of germination [18], determination of the influence of the external environment [19], and specific protein changes [20]. However, little is known about the physiological and molecular adaptive mechanism of wheat plant tolerance to salt in response to PGPR inoculation. Investigation of proteomic profile of wheat plant would give us insights into the molecular mechanism of salt tolerance.

The use of proteomic tools and technologies has facilitated the application of proteomics in the characterization of plant–environmental interactions and should expand our understanding of these processes in the future [21]. The proteomes of plants in response to environmental stimuli have been reviewed previously that illustrated plant proteins are involved in various aspects of plant–bacterial interactions, including plant resistance to pathogenic bacteria, and symbiotic relations for nutrient availability [22]. To date, little work has been done on the combinational effects of plant growth-promoting rhizobacteria and environmental stresses, and corresponding effects on plant proteomes. Thus, the present work was aimed to examine the effect of beneficial PGPR on plant proteomic profile under high salinity stress. Here, a proteomic approach was conducted using Q-TOF (Quantitative time of flight) with column chromatography to explore the alteration in protein expression of wheat plant treated with *Enterobacter cloacae*SBP-8 under salt stress. This approach could be promising to provide new insights into the molecular adaption of plants toward abiotic stressors using a more functional approach.

## Material and methods

### Plant growth and bacterial treatment

The ACC deaminase-containing bacterium *Enterobacter cloacae* SBP-8 was selected for the study based on its plant growth promoting properties under salt-stress conditions [18]. Wheat plants (*Triticum aestivum* C-309) was grown and treated with the test isolate (SBP-8) as described previously with minor modification [23]. Briefly, plant seeds were surface sterilized by treating with 70% ethanol for 2 min followed by three times washing with sterilized water. The seeds were exposed to 1.0% sodium hypochlorite (NaClO) solution for 3 min followed by three consecutive washes using sterile water to remove all traces of bleach solution. The sterilized seeds were treated at room temperature for 1 h with bacterial suspensions (10^8^cfu mL^−1^) of *Enterobacter cloacae*SBP-8following the standard protocol [24]. Sterile 0.03 M MgSO_4_ solution-treated seeds were used as a negative control. For the preparation of bacterial inoculum, the bacterial isolate SBP-8 was grown in 250 ml Luria-Bertani (LB) medium at 30°C for 18 h with continuous shaking. The bacterial cells were harvested by centrifugation at 8,000g for 10 min at 4°C and re-suspended in 25 ml sterilized 0.03 M MgSO_4_ on ice [25]. The absorbance of cell suspension was measured at600 nm, and the turbidity of bacterial suspension was adjusted to a concentration of approximately 10^8^cfu ml^−1^ using sterilized0.03 M MgSO_4_.The sterilized seeds were placed on Petri-dishes containing moistened whatman filter paper with distilled water (10–15 seeds on each plate) for germination. Following germination, five seedlings (3 days old) were sown in each plastic pot filled with sterile soil (400 g). The plants were grown for 15 days after germination under controlled conditions in a plant growth chamber (Labtech, South Korea) set with 340 μmol m^−2^s^−1^ of light for 16:8 h light/dark period at 24±2°C with 70% humidity. The plants were watered with Hoagland medium (pH 6.8) containing NaCl (200 mM) to providing the nutrients as well as imposing the salt stress. Each treatment was taken in triplicate, and pots were arranged in completely randomized block design way. The whole experimental setup was divided into three treatments namely Treatment (T) 1 to 3. T-1 comprised of plants treated with salts and their corresponding untreated control plants. T-2 consisted of plants inoculated with test isolate and their uninoculated control plants, whereas T-3 included inoculated plants treated with salt stress and inoculated control plants grown without salt stress. These plants were grown for 15 days after seed germination, and used for comparison of their proteomic profile. After the experimental period, wheat plants were thoroughly washed with distilled water to remove any adhered soil particles and stored in liquid nitrogen until use.

### Protein extraction

Proteins were extracted using a TCA-Acetone precipitation method with some modifications. The whole plants of wheat (1 g) were finely powdered in liquid nitrogen using a pestle and mortar and suspended in 1 ml of extraction buffer [sucrose0.9 M, Tris-HCl 0.5 M, ethylenediaminetetraacetic acid 5 mM, KCl 0.1 M, and dithiothreitol (DTT) 1% w/v], and vortexed into a thick paste. The suspension was sonicated in an ice-cold sonication bath (4°C) for 5 min in duplicates and was mixed with 1 ml 100% tricarboxylic acid (TCA) and 8 ml 100% ice-cold acetone. The mixture was vortexed for 10 min and centrifuged at 18,000g for 15 min at 4°C. The supernatant was discarded, re-suspended the pellet in 1 ml ice-cold acetone, and washed it at 18,000g for 15 min at 4°C. The above step was repeated to remove all TCA. All acetone was removed, and the sample was dried completely before dissolving in 50mMammonium bicarbonate with 1% SDS. Protein concentration was estimated by Bradford method and 100 μg protein was used for trypsin digestion. The sample was treated with 10mM DTT at 56°C for 1h followed by 55mM iodoacetamide(IDA) at room temperature in the dark for 45min. The sample was then digested with Trypsin (1:100 enzyme/protein concentrations) and incubated overnight at 37°C. The resulting sample was vacuum dried and dissolved in 10μl of 0.1% formic acid in water. After centrifugation at 10000g, the supernatant was injected on C18 Nano-LC column (75μmx150cmx1.7μm BEHC18 column) for separation of peptides followed by analysis on the Waters Synapt G2 Q-TOF instrument (Applied Biosystems, Foster City, CA, USA).

### Liquid chromatography-mass spectrometry analysis

The acquired raw data was processed by MassLynx 4.1 WATERS. The individual peptides MS/MS spectra were matched to the database sequence for protein identification on PLGS (Protein Lynx Global Server) software, WATERS, and MASCOT. The parameters chosen for identification against UNIPROT databases of *Triticum aestivum* included carbamidomethylation of cysteine as fixed modification and oxidation of methionine as variable modification, one missed cleavage, peptide mass tolerance set at 50 ppm (parts per million), fragment mass tolerance set at 0.8 Da, and peptide charges set at +2, +3, and +4.Mouse scoring algorithm was used to derive significance of the protein match with the ion score, which was calculated as -10×LOG_10_(P). The P in above formula represents an absolute probability of the observed match being a random event. It, thus, indicates that the match of a given protein and MS/MS spectra having relatively small P-value is not a random event. Expression of different proteins was compared between different treatments by calculating ratio of peptides in two samples based on ion-abundance data of peptides. Proteins/peptides having ratio of more than 1.5 was considered as up-regulated, whereas values below 0.75 were kept as the threshold for down-regulated proteins/peptides. Differentially expressed proteins were classified according to Gene ontology (http://www.geneontology.org)for their molecular function, biological processes, and cellular processes involved in response to salt stress. According to the known or predicted cellular localization and molecular function of the proteins, as determined by Blast-2Go (http://www.blast2go.com), specific groups of proteins were selected and analyzed on the basis of stimulus responses, chloroplasts proteins, and enzymes.

### Hierarchical cluster analysis of differentially expressed protein

The expression profile of 79 differential expressed proteins (Table 1) common to three tested treatments was constructed through the two-way hierarchical clustering according to the Permut-Matrix software. Rows were mean centered, and Euclidean distance as well as average linkage was used for data aggregation. The customized sets of parameters are employed for the analysis, i.e. low and high expression levels are shown with green and red colors respectively.

**Table 1:** The differentially expressed proteins with their UNIPROT-ID common to selected three treatments (T-1, T-2, T-3) were used for hierarchical cluster analysis displaying differential expression. The customized set of parameters is employed for the analysis, i.e. Low and high expression levels are shown with green and red colors respectively.

UNIQID	Protein Name	Sample Name	T1	T2	T3
A9UL13	Retinoblastoma related protein 1	S1	3.490	0.733	0
B0LXM0	S adenosylmethionine synthase	S2	3.387	0	0.175
B1B5D4	Ninja family protein 2	S3	0.663	0.343	0
B1B5D5	Ninja family protein 3	S4	0.543	0.683	0
B8YG97	Avenin like b11	S5	0.105	0.778	0
B6DZC8	Fructan 1-exohydrolase	S6	0	0.748	0.6831
O04705	Gibberellin 20 oxidase 1 D	S7	0	0.740	0.346
O24473	Eukaryotic translation initiation factor 2 subunit beta	S8	1.616	8.414	0
O64392	Wheatwin 1	S9	0	1.822	0.511
P00413	Cytochrome c oxidase subunit 2	S10	0.360	0.307	0
P05312	NADPH quinone oxidoreductase subunit I chloroplastic	S11	1.733	6.889	0.637
P05151	Cytochrome f	S12	1.803	6.685	0
P08488	Glutenin high molecular weight subunit 12	S13	0.733	0.410	0
P09195	Fructose 1 6 bisphosphatase chloroplastic	S14	0	0.657	0.527
P10387	Glutenin high molecular weight subunit DY10	S15	0.329	0	1.822
P11515	Serine carboxypeptidase 3	S16	0.463	0	0.511
P11534	50S ribosomal protein L2 chloroplastic	S17	0.582	0	0.559
P12112	ATP synthase subunit alpha chloroplastic	S18	1.521	2.181	0.683
P12298	Glucose 1 phosphate adenyltransferase large subunit Fragment	S19	0.543	7.924	0.423
P12300	Glucose 1 phosphate adenyltransferase large subunit chloroplastic amyloplastic fragment	S20	1.858	1.537	3.669
P12782	Phosphoglycerate kinase chloroplastic	S21	0	0.726	4.566
P12783	Phosphoglycerate kinase cytosolic	S22	0	0.582	0.690
P16347	Endogenous alpha amylase subtilisin inhibitor	S23	0	0.711	0.272
P22701	Em protein CS41	S24	0.511	0.755	0.339
P23923	Transcription factor HBP 1b c38	S25	0.145	0.748	0
P25032	DNA binding protein EMBP 1	S26	0.486	0.670	0.070
P26304	NADPH quinone oxidoreductase subunit K chloroplastic	S27	0.343	0.631	1.896
P26667	Ribulose bisphosphate carboxylase small chain PW9 chloroplastic	S28	0.427	2.013	0.740
P27357	Thaumatin-like protein PWIR2	S29	4.349	9.487	0
P27572	NADH ubiquinone oxidoreductase chain 4	S30	0.449	0.733	0
P27736	Granule bound starch synthase 1 chloroplastic	S31	0	0.477	0.307
P27806	Histone H1	S32	1.786	1.716	0.477
P27807	Histone H2B 1	S33	0.631	0	0.718
P30523	Glucose 1 phosphate adenyltransferase small subunit chloroplastic	S34	0.477	0.650	0
P31251	Ubiquitin activating enzyme E1 2	S35	1.552	0.683	0
P31252	Ubiquitin activating enzyme E1 3	S36	1.665	0	0.4538
P33432	Puroindoline A	S37	4.953	0	0.003
P38076	Cysteine synthase	S38	1.716	3.596	0
P46525	Cold shock protein CS120	S39	0	1.803	0.755
P46526	Cold shock protein CS66	S40	0	0.588	2.718
P52589	Protein disulfide isomerase	S41	0.657	0.286	0
P55313	Catalase	S42	0	0.755	0.554
P58311	Photosystem I P700 chlorophyll a apoprotein A1	S43	0	1.698	7.924
P68428	Histone H3 2	S44	0	7.315	0.501
P68538	ATP synthase protein MI25	S45	0.307	1.716	0.463
P69373	Chloroplast envelope membrane protein	S46	84.774	4.789	0.081
P80602	2 Cys-peroxiredoxin BAS1 chloroplastic	S47	0.516	0.600	0.506
P93692	Serpin Z2B	S48	0.165	0	0.711
Q01148	NADH ubiquinone oxidoreductase chain 1	S49	0.323	0.548	0.418
Q02066	Abscisic acid inducible protein kinase	S50	3.781	0.657	0.657
Q02879	TATA box binding protein 2	S51	0.326	4.481	0
Q03033	Elongation factor 1 alpha	S52	0	0.637	1.973
Q03968	Late embryogenesis abundant protein group 3	S53	0	0.644	0.677
Q1W374	Phosphomannomutase	S54	0.018	0.697	0.261
Q1XIR9	4 hydroxy 7 methoxy 3 oxo 3 4 dihydro 2H 1 4 benzoxazin 2 yl glucoside beta D glucosidase 1a chloro	S55	0.726	0	0.644
Q2QKB3	Splicing factor U2af large subunit A	S56	0.690	0	0.307
Q2UXF7	Fructan 6 exohydrolase	S57	0	0.367	0.748
Q41593	Serpin Z1A	S58	0	1.993	1.822
Q43206	Catalase 1	S59	0.677	1.568	0
Q43215	Histone H2B 4	S60	0.179	0	0.733
Q43217	Histone H2B 3	S61	0.453	0	0.733
Q43691	Trypsin alpha amylase inhibitor CMX2	S62	0.246	1.733	1.803
Q41558	Transcription factor HBP 1b c1 Fragment	S63	2.095	0	0.600
Q5I7K9	60S ribosomal protein L30	S64	17.993	0	0.027
Q6W8Q2	1 Cys-peroxiredoxin PER1	S65	0.650	0	0.733
Q84N28	Flavone O-methyltransferase 1	S66	0.436	0	0.650
Q84N29	Probable non specific lipid transfer protein 3	S67	0.511	0.625	0.472
Q8LK61	NADP dependent glyceraldehyde 3 phosphate dehydrogenase	S68	0	1.552	0.058
Q95H42	NADPH quinone oxidoreductase subunit H chloroplastic	S69	0	0.496	0.554
Q95H43	NADPH quinone oxidoreductase subunit 1 chloroplastic	S70	7.845	0.763	0
Q95H53	30S ribosomal protein S11 chloroplastic	S71	3.819	0.477	0
Q9S7U0	Inositol 3 phosphate synthase	S72	0.543	0.548	0
Q9XPS6	Photosystem II reaction center protein M	S73	0.117	0	0.440
Q9XPS9	DNA directed RNA polymerase subunit beta	S74	0.726	0.718	0
Q9ZRB1	Tubulin beta 2 chain	S75	0	0.748	0.733
Q9ZRB7	Tubulin alpha chain	S76	0.594	0	1.537
Q9XPS8	Photosystem II CP43 reaction center protein	S77	0	0.670	0.600
Q9SWW5	Glutathione gamma glutamyl cysteinyltransferase 1	S78	0	0.755	0.254
Q9ST58	Serpin Z1C	S79	0	2.075	0.588

## Results

### Proteomic analysis

**Fig 1:**
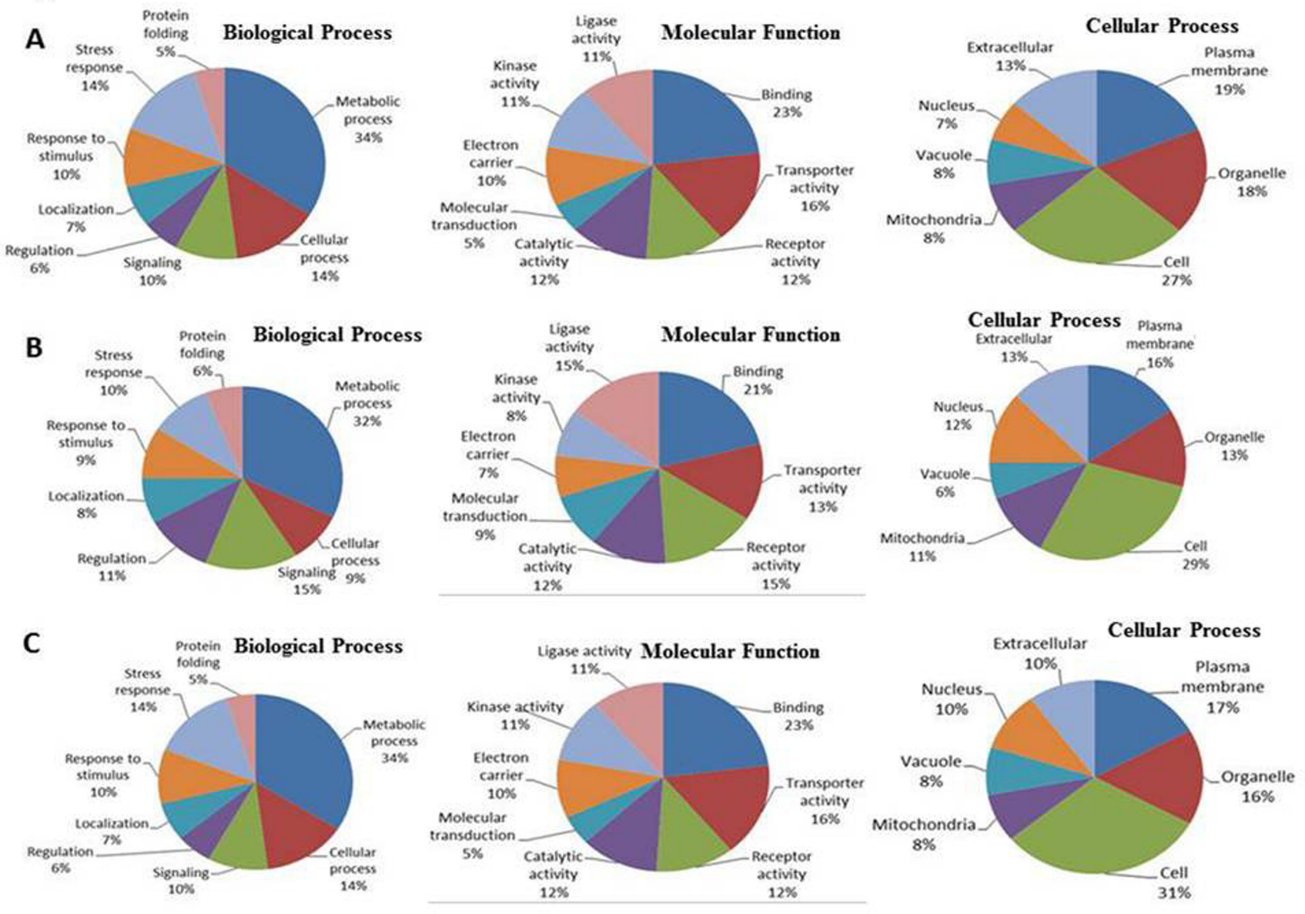
Pie charts showing the distribution of differentially expressed proteins based on their predicted biological process, molecular functions, and cellular process in: (A) Treatment T-1, (B) Treatment T-2, and (C) Treatment T-3.

As in every treatment for protein comparison, two sets of plants were taken. In the T-1 treatment (uninoculated control plants treated with and without salt stress), a total of 301 proteins were identified. Similarly, in the T-2 (bacterial inoculated plants and their uninoculated control plants), and T-3 treatment (bacterial inoculated plants treated with salt stress and bacterial inoculated control plants grown without salt stress) a total of 307 and 286 protein were identified respectively (S1 File).A total of 278 proteins were common to T-1 and T-2 treatment, whereas 266 and 243 proteins were common between T-1 and T-3, and T-2 to T-3.To study the effects of bacterial inoculation and salinity effect, different levels of protein expression and differentially accumulated proteins were assigned to major functional groups, and it was further subcategorized for greater clarity (Fig 1). All identified proteins were classified by gene ontology (GO) annotation software and then classified into three functional groups: molecular function, biological process, and cellular component. The results of the GO analyses for the various treatments are shown in Fig 1. Most of the annotated molecular functions were found to relate to binding, transporter and receptor activity, while most of the annotated biological processes were found related to metabolic and signalling processes.

Among the differentially expressed proteins categorized under biological process, 25proteinswereclassifiedas binding proteins for DNA, protein, or nucleotide, eighteen were involved in transporter activity, and 13were classified as receptor proteins in T-1 experimental plants (Fig 1). Proteins related to stress responses (12), regulation (8) and signal transduction (13) were also identified. For the T-2 treatment, 15 proteins were categorized for receptor activity, thirteen was involved in the transporter, and 21 were classified as binding proteins for DNA, protein, and nucleotide (Fig 1). Bacterial inoculation slightly increases the regulatory (11) and signal transduction (15) proteins. For the T-3 treatment, 23 were recognized as binding proteins, 16 for receptor activity, and 12 for catalytic activity (Fig 1)

### Functional annotation and classification of identified proteins

The fifteen functional groups included the proteins involved in cell division, chromatin-associated protein, defense and pathogenic protein, ion transport, lipid biosynthesis, metabolic pathway, photosynthesis, plant growth and development, protease inhibitor, protein synthesis, protein degradation, seed storage, stress-related, transcription control, and some others with known biological functions. The majority of the identified proteins were related to a metabolic pathway, photosynthesis, and stress mechanisms.

**Fig 2:**
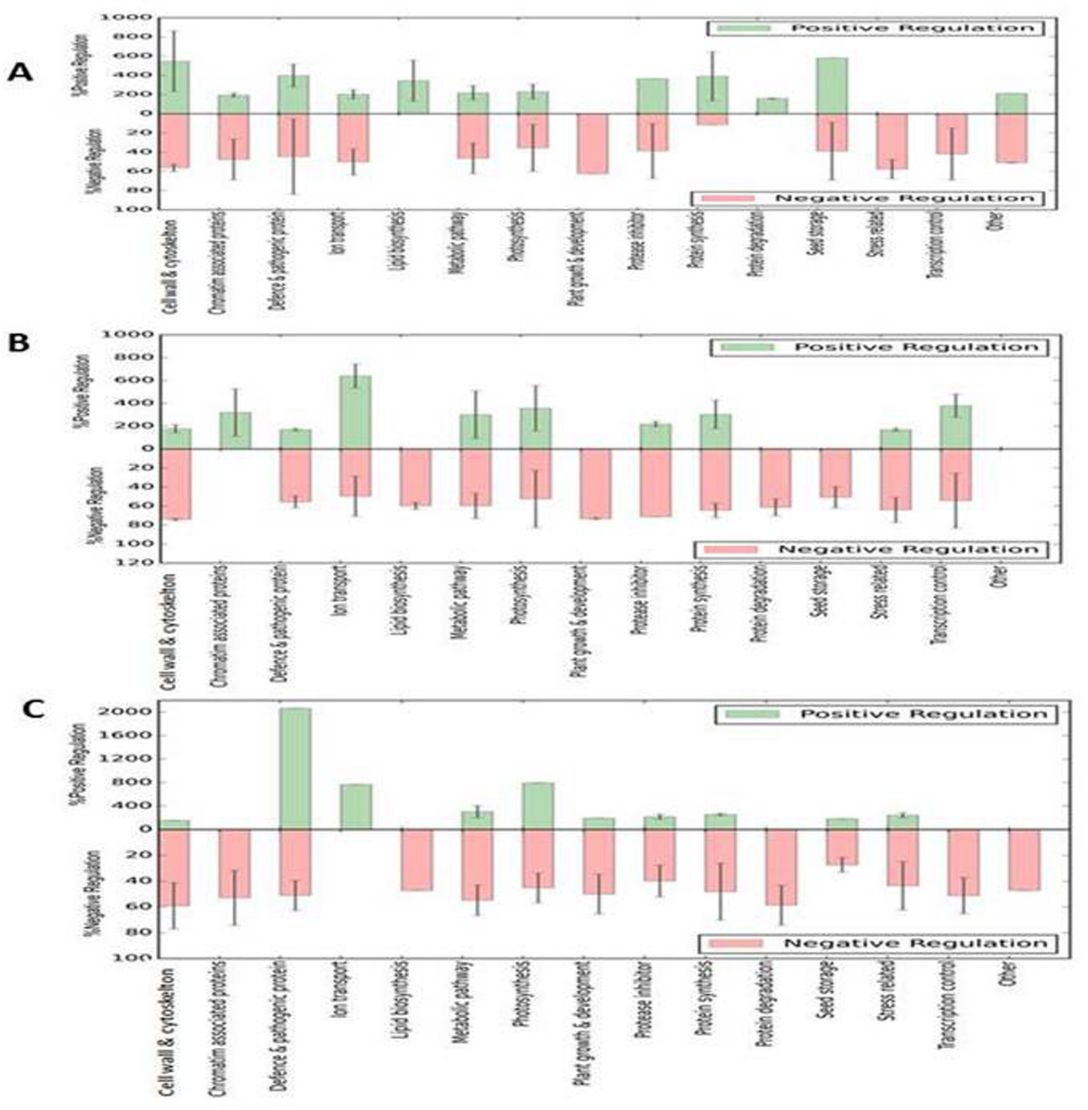
The major functional groups of proteins identified in treatment: (A) T-1 (control vsuninoculated plants with salt stress), (B) T-2 (control vs inoculated plants), and -3 (bacteria- inoculated vs salt stress). The positive regulation represent the >1.5 fold ratio level of expression of that protein, whereas negative regulation showing <0.75 fold ratio of expression. Standard deviation (SD) in each functional category was calculated by measuring the expression level of the entire proteins in given category.

Following bacterial inoculation, the major changes occurred for the proteins involved in primary metabolism and stress mechanisms. Bacterial inoculation up-regulates the level of the proteins involved in defense, a protease inhibitor, and protein synthesis. In the presence of NaCl treatment, the level of proteins involved in cell division, defense, protein synthesis and stress-related were down-regulated. The highest protein up-regulation was observed for seed storage protein (581%), followed by the protein related to cell division (547%) in treatment T-1 (Fig 2). It is evident from Fig 2 that salt stress down-regulated proteins related to plant growth and development,stress proteins, and transcriptional control by 62%, 57%, and 42% respectively.

In response to bacterial inoculation (T-2), the highest increase in up-regulation of protein was observed for ion transport (640%), transcriptional control (379%), photosynthesis (356%), and chromatin-associated protein (319%) (Fig 2). Besides, the down-regulated protein belonged to lipid biosynthesis (60%), plant growth and development (73%), protein degradation (61%) and stress-related protein (51%) (Fig 2). In other treatment (T-3), it was observed that proteins related to defense response were up-regulated by (2065%), photosynthesis (792%), and ion transport (765%). The down-regulated proteins were chromatin-associated protein (53%), lipid biosynthesis (47%), protein degradation (58%), and transcriptional control (51%) (Fig 2)

**Fig 3:**
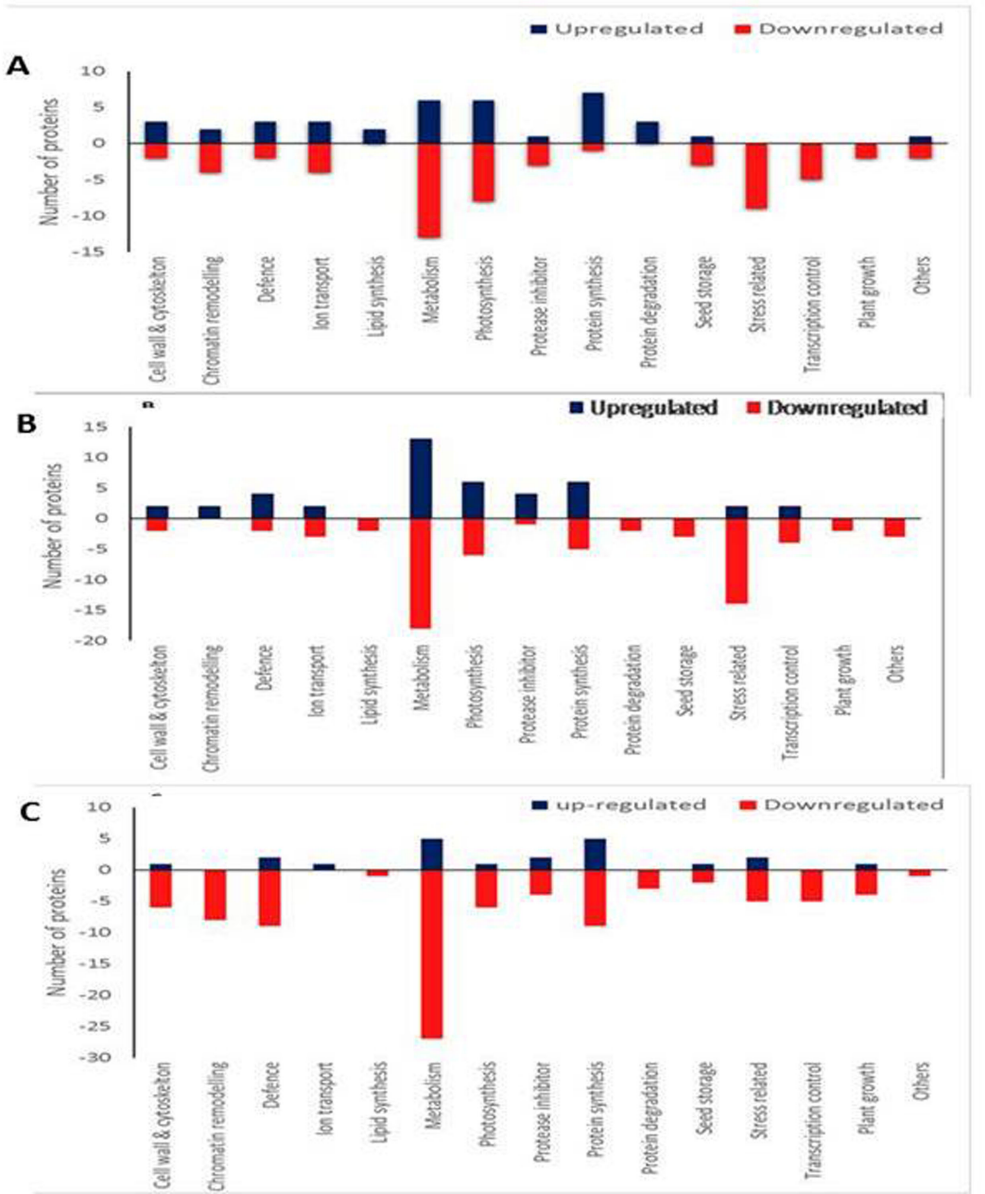
Number of proteins up-regulated/down-regulated in each functional category in treatment: (A) T-1, (B) T-2, and (C) T-3.

Following expression analysis, based on significant changes of ≥ 1.5-fold or ≤ 0.75, proteins belonging to different category were differentiated. Among 96 proteins that showed predominant changes in treatment T-1, 38 were up-regulated, and 58 were down-regulated (Fig 3). The higher increase in protein number was observed for protein synthesis (7), followed by photosynthesis (6) and metabolism (6). In response to bacterial inoculation (T-2), the expression level of 110 proteins showed significant changes, of which 43 were up- regulated, and 67were down-regulated (Fig 3). The metabolic proteins (13) were observed in higher number as compared to others. The expression level of 111 proteins showed significant changes in treatment T-3, of which 21 were up-regulated, and 90 were down-regulated (Fig 3). The proteins related to metabolic and protein synthesis (5) was predominantly present as compared to others.

### Identification of differentially expressed proteins

**Fig 4:**
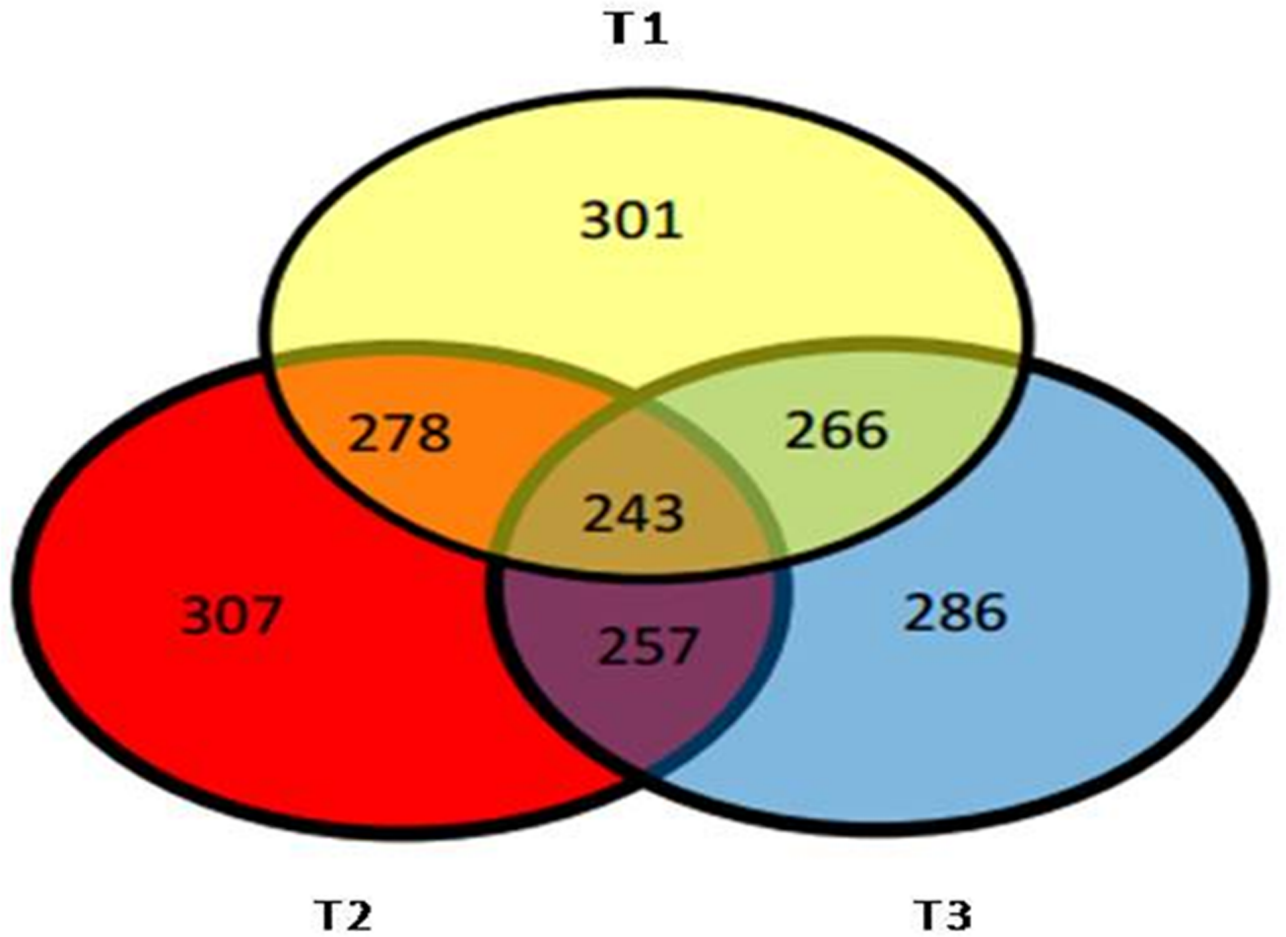
Venn diagram representing the presence and common proteins in each treatments (T-1, T-2, T-3).

To depict the common and unique proteins identified in each treatment, we constructed the Venn diagram, where the numbers of proteins in response to bacterial inoculation and NaCl stress are reported. A total of 301 proteins observed in treatment T-1 (control vs. control with salt stress), 307 in treatment T-2 (control vs bacterial inoculated), and 286 in treatment T-3 (bacterial inoculated vs. bacterial inoculated with salt stress). After comparison, 278proteins were common in treatment T-1& T-2, whereas 256 and 266proteins were common to treatment T-1 & T-3, and between T-2 &T-3 respectively (Fig 4). Among the identified proteins, 243 proteins were identified under all treatments.

### Cluster analysis of differentially expressed proteins

**Fig 5:**
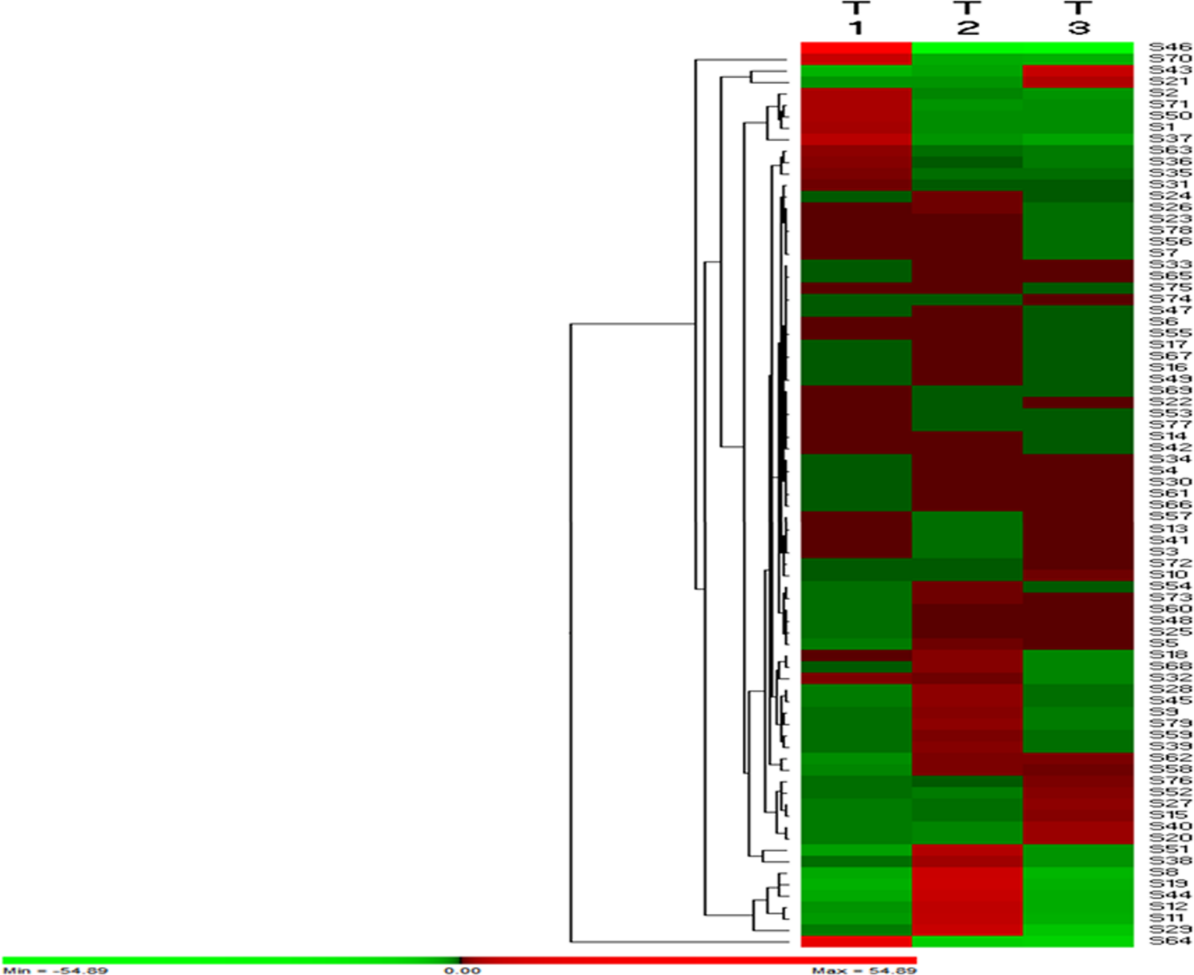
Hierarchical cluster analysis of 79 differentially expressed proteins common to the three experimental treatments (T-1-T-3). T-1 treatment represents the untreated control plant versus salt stress (200 mM NaCl). T-2 treatment represents the control plant against the bacteria inoculated plant. T-3 is the comparison against bacterial inoculated plant against salt stress in the presence of bacterial inoculum. Up-regulation or down-regulation is indicated by the green and red color respectively. The intensity of the colors increases as the expression differences increase, as shown in the bar at the bottom.

A total of 79 differentially expressed proteins common to all treatments (T-1, T-2, T-3) were used for hierarchical cluster analysis under different treatments (Table 1) protein with UNIPROT-ID). These 79 proteins were chose based on their higher abundance. In the T-1 treatment, the cluster contained the 19 up-regulated and 38 down-regulated proteins. Examples of proteins that were majorly decreased under salt stress are Photosystem I chlorophyll a (S 43), Trypsin alpha-amylase inhibitor (S 62), Glucose-1-phosphate adenyltransferase (S 19) and Histone (S 44). The other down-regulated protein was belonging to Peroxiredoxin protein (S 65), Ribulosebisphosphate carboxylase (S 28), and ATP synthase protein (S 45). Bacterial inoculation enhanced the expression of the proteins belonging to ATP synthase (S 18, 45), Ribulosebisphosphate carboxylase (S 28), Translation initiation factor (S 8), Glucose 1 phosphate adenyltransferase (S 19), and Histone H3 (S 44) under non-saline stress. Under the salt stress of 200 mM, bacterial inoculation enhanced the expression of Phosphoglycerate kinase (S 21), Photosystem I (S 43), Glucose-1- phosphate adenyltransferase (S 20) and Cold shock protein (S 40). The down-regulated proteins were belonging to Chloroplast envelope membrane protein (S 46), NADP-dependent glyceraldehyde 3 phosphate dehydrogenase (S 68), Thaumatin-like protein (S 29), and Ribosomal protein (S 64) (Fig 5).

## Discussion

Previous studies have demonstrated that inoculation of PGPR can up-regulate or down-regulate expression of the proteins in response to salinity and other stresses [26,27]. In this study, the variation in expression of the proteome of wheat plants due to NaCl treatment was analyzed in plants inoculated with PGPR *Enterobacter cloacae* SBP-8. It is well documented that on exposure to different stresses such as temperature, drought, and salinity, plants develop one or several other mechanisms which are regarded as adaptive mechanisms to sustain in given stress conditions. These adaptive features (modifications) can be exhibited at genetic, molecular, membranous and cellular levels. These modifications can occur either at one or many levels as mentioned above. The present study demonstrated that plants possess several different adaptive mechanisms in response toNaCl stress to cope with imposed stress by regulating the genes for proteins involved in stress, photosynthesis, transcriptional control, and protein synthesis [27]. This altered gene expression in response to NaCl stress results in up-regulation/down-regulation of various stress-related proteins and protects the plant from stress. Thus, an understanding of these differential expressions could provide an insight into plant’s response to NaCl stress. In the present study, we used the gel-free proteomics protocol for the identification of proteins, as it allows major changes and even deeper analysis of the proteome. The functional groups related to different categories are discussed in detail in following sections.

### Cell structure maintenance, cell division, and chromatin-associated protein

Maintenance of cellular integrity is of paramount importance for the organisms thriving in high osmotic stress conditions. In the present study, this was supported from the up-regulation of few cytoskeletal proteins such as ‘Tubulin’ which is required for the maintenance of cell integrity [28]. Protection of cell integrity under salt stress was also guaranteed by the increase in the levels of ‘Profilin’ that binds to actin and affects the structure of the cytoskeleton. Another up-regulated gene was one encoding ‘Retinoblastoma’ which is involved in cell-cycle progression, endoreplication, transcriptional regulation, chromatin remodeling, and cell growth were observed in the bacterial inoculated plants.

Bacterial inoculation enhanced the expression of ‘Casparian strip membrane protein’(CASP) and ‘Xyloglucanendo transglycosylase (XET)’ as compared to uninoculated plants treated with salt stress. CASP regulates membrane-cell wall junctions and prevents lateral diffusion of molecules by recruiting the lignin polymerization machinery in the endodermis [29], whereas XET relegates xyloglucan polymers, an essential constituent of the primary cell wall, and thereby participates in cell wall construction of growing tissues. In spite of the critical role of the endodermis development, very little is known about the biosynthetic mechanism of Casparian strip formation. XET integrates the newly secreted xyloglucan chains into an existing wall-bound xyloglucan restructuring existing cell wall material by catalyzing transglycosylation between previously wall bound xyloglucan molecules [30]. Sometimes XET act as hydrolase (XEH), hydrolyzing one xyloglucan molecule, depending on the nature of the xyloglucan donor and acceptor substrates [31,32]. Furthermore, XET may be important for regulating the polymer length and insertion of xyloglucans into the cell wall, which could alter the extensibility of the cell wall [31]. A decrease in XET activity was reported in primary roots of maize with low water potential, which was correlated with a decrease in cell wall extensibility and cell elongation in that region [33]. In contrast to this, enhanced XET activity under abiotic stress like drought and heat in durum wheat was correlated with an increase in cell wall extensibility [34, 35].

### Ion-transporters and lipid biosynthesis

In response to bacterial inoculation, the differential level of expression of iontransporter proteins was observed under salinity stress. In the ion-transporter category, four proteins were down-regulated under 200 mM NaCl stress with respect to the control and three proteins were up-regulated. As compared to control, bacterial inoculation enhanced the expression of ‘Malate transporter’ and ‘Two pore calcium channel protein’, whereas down-regulates the expression of ‘Mitochondrial outer membrane porin’. At high salinity stress of 200 mM NaCl, bacterial inoculation enhanced the ‘calcium channel protein’ that acts as the major ROS-responsive Ca^2+^ channel and mediates the salinity -induced Ca^2+^ influx in leaf cells. Ca^2+^ acts as the second messenger in response to environment stimuli under salt stress. A diversity of Ca^2+^ responsive proteins facilitates the regulation of their target proteins by coordinating the diverse signaling pathways [36]. It has been demonstrated in an earlier study that the induced expression of calcium channel protein plays a pivotal role in regulating calcium homeostasis and protein folding in the endoplasmic reticulumin rice leaves under osmotic stress [37]. Therefore, signaling pathway mediated by calcium seems to be an important strategy of wheat seedlings in coping with salt stress.

The protein involved in the lipid biosynthesis provides the stability of cellular membranes and increases the ability to bind and/or carry hydrophobic molecules across the membrane [38]. Various lipid biosynthesis proteins such as ‘Obtusifoliol 14 alpha-demethylase’ (CYP51) and ‘Puroindoline’ were up-regulated at 200 mM NaCl with respect to the bacterial inoculation. CYP51 are involved in the steroid biosynthesis pathway, whereas ‘Puroindoline’ forms monovalent cation-selective ion channels in membranes and also act as membrane toxin to protect the plants against predators. CYP51 is the most widely distributed cytochrome P450 gene family being found in all biological kingdoms. It catalyzes the first step following cyclization in sterol biosynthesis, leading to the formation of precursors of steroid hormones, including brassinosteroids in plants [38]. The increase in the sterol/brassinosteroids is essential for plant growth and reproduction. Inaddition, these are capable of increasing plant tolerance to both biotic stresses like pathogen attack and abiotic stress like drought, salinity, and heatetc. [39].

Bacterial inoculation enhances the ‘Non-specific lipid transfer protein’ (nsLTPs) expression that transfers phospholipids as well as galactolipids across membranes and plays a role in wax or cut in deposition in the cell walls of expanding epidermal cells and certain secretory tissues. Then sLTPs are small, basic proteins present in abundance in higher plants. They are involved in key processes of plant cytology, such as the stabilization of membranes, cell wall organization, and signal transduction. These are also known to play important roles in resistance to biotic and abiotic stress and in plant growth and development, such as sexual reproduction, seed development and germination [40]. Although nsLTPs have been extensively studied, their modes of action in intact cells have not yet been fully elucidated.

### Defense/Stress-related proteins (including pathogenesis protein also)

The data showed that few of the defense proteins such as Clp protease, Thioredoxin H, 2 Cys-peroxiredoxin, Catalase and Ninja family protein were down-regulated at 200 mM NaCl as compared to control plants. However, in the presence of bacterial inoculation, few of the defense related proteins belonging to Ninja family were up-regulated under salt stress. The increase in accumulation of defense proteins could be due to increase in the expression of these proteins in bacteria inoculated plants. Clp protease shows the chymotrypsin-like activity and plays a major role in the degradation of misfolded proteins, but a physiological role in plants has not been well established yet. Thioredoxin H probably behaves as an antioxidant enzyme particularly important in the developing shoot and photosynthesizing leaf under stress. 2 Cys-peroxiredoxinmay be an antioxidant enzyme particularly important in the developing shoot and photosynthesizing leaf. Catalase is an antioxidant enzyme involved in different processes such as H_2_O_2_ detoxification, stress response, and senescence [41].

The role of Ninja family protein in the plant has not been well understood. However, it is postulated that it acts as a negative regulator of abscisic acid (ABA) response during germination through the ubiquitin-mediated proteolysis of ABI5/DPBF1. Bacterial inoculation up-regulates the expression of Transcription factor HBP 1a and Cold shock protein CS120 (belong to ninja family of proteins) as compared to uninoculated plants. HBP 1a is a putative transcription factor which regulates histone gene expression. Cold shock protein CS120 may reduce intracellular freezing damage during winter by hydrogen-bonding to the lattice of the nascent ice crystals, thus modifying the structure and/or propagation of ice crystals. In addition, inoculation of bacterium up-regulated the expression of Hsp70, Hsp90 organizing protein, and Cold shock protein CS66 at 200 mM NaCl stress, as compared to control plants with respective salt stress. Many of the other stress-related proteins were down-regulated, illustrating that in the presence of bacterial inoculums, plants did not face the stress conditions.

Besides these, other proteins related to pathogenesis such as Thaumatin-like protein (TLPs), Alpha amylase trypsin inhibitor, Purothionin, Puroindoline B, Wheatwin, and Serpin Z1A were found to be up-regulated in the presence of bacterial inoculation under salt stress (T-3). The expression of these proteins provides insight into the understanding of cross-tolerance mechanism in wheat plants in response to biotic and abiotic stress. The pathogenesis-related protein plays a crucial role in response to pathogens. However, their involvement in salinity stress has been demonstrated in several crops [42,43]. TLPs are reported to be widely distributed PR proteins across kingdoms including gymnosperm, angiosperm, and have been isolated and characterized from different plants and tissues. These are involved in the formation of disulfide linkages, which impart stability to the protein under varied thermal and pH conditions and are shown to be involved effectively in alleviating both biotic and abiotic stress tolerance [44,45]. Alpha amylase trypsin inhibitor could be involved in insect defence mechanisms.

Purothionin in conjunction with thioredoxin, affects proper protein folding, cytotoxic, presumably by forming pores in the cytoplasmic membrane. Their precise function is not known. Puroindoline B has antimicrobial activity against several bacterial and fungal pathogens. Wheatwins are pathogenesis-related proteins of the PR-4 family and shows antifungal activity towards the wheat-specific pathogenic fungi *Fusarium culmorum* and *F*.*graminearum*. In addition, wheatwin has been demonstrated to possess an RNAse activity that may be part of a mechanism for inhibiting invading pathogens [46]. The expression of wheatwin genes has been investigated in a number of tissues, particularly in response to pathogen challenge [47]. However, the potential role of wheatwinin response to abiotic stress has not been investigated. The wheat serpins are suicide substrate inhibitors of chymotrypsin and cathepsin-A that may serves to inactivate serine proteases of grain-boring insects [48]. They have not yet been assigned to specific genetic loci on the wheat chromosomes. Additionally, in presence of bacterial inocula decrease in the expression of Glutathione–S-transferase (GST) was noted.

### Protein synthesis/degradation

Increase in NaCl stress causes a significant suppression of protein synthesis and its intermediate pathways [49]. We found that levels of Elongation factor 1 and Protein disulfide isomerase were decreased at NaCl, whereas activation of Ubiquitin-activating enzyme E1 1, E1 2, E1 3, Eukaryotic translation initiation factor 2, Translation initiation factor IF 1 and Ribosomal protein were increased. Activation of ubiquitin enzymes illustrates the proteolytic activity in response to NaCl stress. Many of the enzymes responsible for protein degradation and proteolytic complexes involved in recognizing and removing abnormal proteins were found to be up-regulated at NaCl stress. Ubiquitin enzymes play a central role in metabolism under abiotic stress as they are involved in protein inactivation, degradation of damaged proteins, and release of amino acids for metabolism [50]. The levels of various ribosomal subunits were differentially decreased with respect to salt stress. Nevertheless, eukaryotic translation initiation factor 4B1, 4E 1 and 2 were significantly increased in response to bacterial inoculation. Therefore, it can be assumed that regulation of the translational machinery is an important component of stress response in plants [51].

### Photosynthesis

Photosynthesis is one of the physiological processes that are very sensitive to salt stress. The primary effect of salt stress is the reduction of stomatal aperture in leaves, which leads to the reduction of CO_2_availability and thus minimizes the energy for plant growth. We found a significant increase of the level of Ribulose-1,5-bisphosphate carboxylase oxygenase (RuBisCO) large subunit at 200 mM NaCl. The increase in the levels of RuBisCO subunits seems to partially offset the energy reduction that naturally occurs under salinity stress [51]. RuBisCO is the key enzyme for CO_2_ assimilation and catalyzes the carboxylation of D-ribulose 1,5-bisphosphate, the primary event in CO_2_ fixation in the Calvin cycle. It is stroma-localized protein and constitutes up to 50% of all chloroplast proteins. Salt stress increases oxidation and decreases carboxylation activities of RuBisCO, and causes to decrease in severity of CO_2_ fixation [52].

The other enzymes that increased included Cytochrome f, Photosystem II protein D1, Cytochrome b, Photosystem II CP47 reaction center protein, and Chloroplast envelope membrane protein. Cytochrome f mediates electron transfer between Photosystem II (PSII) and Photosystem I (PSI) as well as cyclic electron flow around Photosystem I (PSI) [53]. Photosystem II protein D1 forms the reaction core of PSII as a hetero-dimer with the D2 protein and withdraws electrons from water, leading to the splitting of water and the formation of molecular oxygen. Cytochrome b is the component of the ubiquinol-cytochrome c reductase complex (complex III or cytochrome b-c1 complex), which is a respiratory chain that generates an electrochemical potential coupled to ATP synthesis [54]. Photosystem II CP47 reaction centreproteinsare the intrinsic transmembrane antenna proteins CP43 (PsbC) and CP47 (PsbB) found in the reaction centre of PSII, to cope with light limitations and stress conditions. The molecular function of Chloroplast envelope membrane protein (CemA) is unknown, however partially, involved in light-induced Na^+^-dependent proton extrusion and has been implicated in CO_2_ transport [55]. We found various differentially expressed proteins related to energy and metabolism after NaCl stress in the presence of bacterial inoculation. The down-regulated proteins belonging to photosystem category were of Chlorophyll a, b binding protein, Cytochrome b6, Photosystem II reaction centre protein M, Ribulosebisphosphate carboxylase small chain PW9, Cytochrome b6 f complex iron-sulfur subunit, Photosystem I P700, Photosystem II, and 50S Ribosomal protein.

### Proteins of metabolism

Several differentially expressed proteins related to metabolism were observed. Proteins those were up-regulated were as follows: Imidazole glycerol phosphate dehydratase, Fructan-1-exohydrolase, Fructan-6-exohydrolase, Fructan-1-exohydrolase, NADPH quinoneoxido-reductase, adenosyl-homocysteinase, andGlucose-1-phosphate adenylyltransferase, and Phosphoglycerate kinase. The up-regulation of gene for the synthesis of Fructan-1-exohydrolase seems obvious, as apart from encoding storage compounds, fructans might have an important role in protection against stress [56]. These accumulated low molecular weight water-soluble compounds are known as “compatible solutes” or “osmolytes” is the common strategy adopted by several organisms to combat the environmental stresses. Osmotic adjustments at physiological levels are keys to survive under salinity stress. The osmoregulators protect plant cells during extreme stress conditions of salt. Furthermore, genes related to osmoprotectant biosynthesis are up-regulated under salinity stress [57]. Also, their accumulation is preferentially favoured under salt stress because they provide tolerance against stress [58]. The level of accumulation of osmo-protectants in different species provides different levels of protection during abiotic stresses. Genes responsible for the synthesis of different types of compatible solutes have been isolated from various organisms. Genetic engineering attempts are being made with these endogenous or ectopic genes to successfully use to synthesize compatible solutes in target organisms to improve stress tolerance [59,60].

### Role of differentially expressed proteins

In response to salt stress and bacterial inoculation, the level of ATP synthase that is primarily involved in the energy production processes was enhanced. Previous study has shown the positive association between the defense response and primary metabolic process involved in energy production like ATP biosynthesis, pentose phosphate pathway, TCA cycle, electron transport, and glycolysis [61]. The complexity of plant defense responses requires abundant amount of energy. In addition, the primary metabolic pathways play a role as a source of signaling molecules to trigger defense responses [62]. Based on the observed results, it is plausible that expression of metabolic proteins like ATP synthase serves as a switch to turn on or off the different connections between carbohydrate metabolism and defense responses.

Similarly, bacterial inoculation enhanced the expression of Ribulose bisphosphate carboxylase that are related to the increase of availability of photosynthates/carbon skeletons both for biosynthetic reactions and production of energy. Previous study has demonstrated that PGPR *Pseudomonas fluorescens* KH-1 up-regulated the expression of Ribulose bis phosphate carboxylase which is an important enzyme in chloroplast metabolism and photosynthesis. The increased level of Phosphoglycerate kinase catalyzes the formation of ATP from ADP in glycolysis where 1,3-bisphosphoglycerate is converted to 3-phosphoglycertae. This reaction is essential in most cells for ATP generation and for carbon fixation in plants. However, under stress conditions, do plants need to energy has not yet been reported. In addition, bacterial inoculation enhanced the Glucose-1- phosphate adenyltransferase expression, that have positive consequences on plant growth and metabolism both when plants is in physiological condition and even more when plants are under stress conditions [63].

## Conclusions

Based on protein profiling using gel-free method, the present study reports the amelioration of salt stress in wheat by a plant growth promoting bacterium *Enterobacter cloacae* SBP-8 which modulates expression of different proteins involved in maintenance of cell structure, division, protein synthesis, proteolysis, photosynthesis, defense, fatty acid synthesis, homeostasis, and other metabolic pathways. Inoculation of SBP-8 upregulates expression of proteins such as tubulin, profilin, retinoblastoma, CASP (casparian strip membrane protein), xyloglucan endotransglycosylase etc., which can play eminent role in strengthening of cell wall and maintenances of cell structure to prevent cellular damage during salt stress condition. It also modulates expression of membrane ion-transporter proteins including malate transporter, and ROS-responsive calcium channel proteins which are pivotal in maintenance of ion-homeostasis to cope with salt stress. The bacterial inoculation increased expression of proteins and non-specific lipid transfer proteins involved in biosynthesis of lipids such as obtusfoliol, and steroid hormones. It further enhanced expression of Clp proteases, thioredoxin H, catalase, proteins of Ninja family, thaumatin like proteins, and other defense-related enzymes/proteins which indicate the overlap in mechanisms of plant responses to biotic and abiotic stressors. PGPB-mediated suppression of protein synthesis pathway, ribosomal subunits, and activation of ubiquitin activating enzymes suggest the possible strategy for the abetment of damages caused by salt stress. Finally, the bacterial inoculation also upregulates the proteins involved in metabolic pathways leading the synthesis of storage proteins and osmoprotectants which are the most common protective mechanisms to overcome growth inhibition caused by salt stress. Thus, the present study provides evidence that the application of a beneficial PGPR to wheat seedlings could be used as an effective tool to overcome the salinity stress. The identified proteins can be useful for genetic transformation to improve the salt tolerance mechanism in wheat-like cereal crops. The future study should be directed to quantitatively analyze the differentially expressed proteins under different salinity treatments to elucidate the proper salt-tolerance mechanism.

## Supporting information

S1 File The total protein recorded in the T-1 treatment (uninoculated control plants treated with and without salt stress), T-2 (bacterial inoculated plants and their uninoculated control plants), and T-3 treatment (bacterial inoculated plants treated with salt stress and bacterial inoculated control plants grown without salt stress). (XLSX)
